# Healing the Past by Nurturing the Future: Aboriginal parents’ views of what helps support recovery from complex trauma: Indigenous health and well-being: targeted primary health care across the life course

**DOI:** 10.1017/S1463423621000463

**Published:** 2021-09-30

**Authors:** Catherine Chamberlain, Yvonne Clark, Stacey Hokke, Angela Hampton, Caroline Atkinson, Shawana Andrews

**Affiliations:** 1 Judith Lumley Centre, School of Nursing and Midwifery, La Trobe University, Melbourne, Victoria, Australia; 2 NGANGK YIRA: Murdoch University Research Centre for Aboriginal Health and Social Equity, Murdoch University, Perth, Western Australia; 3 SAHMRI Women and Kids Theme, South Australian Health and Medical Research Institute, Adelaide, South Australia; 4 School of Psychology, University of Adelaide, Adelaide, South Australia; 5 Central Australian Aboriginal Congress, Alice Springs, Northern Territory, Australia; 6 We Al-li Pty Ltd, Goolmangar, New South Wales, Australia; 7 School of Health Sciences, University of Melbourne, Melbourne, Victoria, Australia

**Keywords:** Aboriginal and Torres Strait Islander, complex trauma, Indigenous, parent, views

## Abstract

We aimed to understand support needs for Aboriginal and Torres Strait Islander parents experiencing complex trauma.Becoming a parent is an exciting yet challenging transition, particularly for parents who have experienced past hurt in their own childhood which can have long lasting effects, including complex trauma. Complex trauma-related distress can make it harder to care for a baby, but the parenting transition offers unique opportunities for recovery.

This formative research is part of a community-based participatory action research project which aims to co-design perinatal awareness, recognition, assessment and support strategies for Aboriginal and Torres Strait Islander parents experiencing complex trauma. We used an Indigenist approach and grounded theory methods. Aboriginal and Torres Strait Islander parents who were pregnant and/or have children up to two years old were recruited through perinatal care services and community networks in three Australian sites (Alice Springs, Adelaide and Melbourne). Parents were offered a group discussion or individual interview, facilitated by Aboriginal researchers. Third-person scenarios and visual tools were used to facilitate reflections about the impact of past experiences, what keeps parents strong, hopes and dreams, and what is needed to achieve those dreams. Parents were also shown themes from a previous systematic review of parents’ experiences as a prompt to identify any additional key issues.

Seventeen Aboriginal and Torres Strait Islander parents participated in August to September 2019. Most were mothers (n = 15).  The study’s grounded theory methods provided the foundation of a theoretical supposition that positions the transformation of the compounding cycle of trauma, to a reinforcing cycle of nurturing at the intersection of: 1) parents’ connectedness; 2) social and emotional wellbeing; and 3) the transition to parenting. Unique opportunities and challenges situated at the interface are bound to the compounding or reinforcing nature of the intersecting factors. Findings reveal complexity, differing experiences by gender and age, as well as within and between communities.

## Introduction

Becoming a parent is an exciting transition, but it can be challenging, particularly for parents who have experienced maltreatment in their own childhoods (Chamberlain *et al.*, 2019a). Childhood maltreatment, affecting up to 50% of all children worldwide, can have profound and ongoing impacts on development and physical, social and emotional well-being. These effects can include complex post-traumatic stress disorder (complex trauma) (Böttche *et al.*, 2018). Features include *emotional dysregulation*, *negative self-concept* and *relational disturbances*, *re-experiencing events (triggers)*, *avoidance* and a *sense of threat* (Cloitre *et al.*, 2014).

Aboriginal and Torres Strait Islander communities are particularly affected by complex trauma following a legacy of historical trauma which included state-sanctioned removal of Aboriginal and Torres Strait Islander children from their families and ongoing discrimination (Atkinson, 2002). Successive waves of violence since occupation have disrupted family and community networks that for millennia centred on fostering social and emotional well-being, consistent with Aboriginal and Torres Strait Islander worldviews of connectedness and relatedness (Gee *et al.*, 2014). The World Health Organization provides a framework (Marmot *et al.*, 2012) for understanding how the compounding intergenerational effects of complex trauma impact the health of Aboriginal and Torres Strait Islander communities (Chamberlain *et al.*, 2019a). These include historical violence which has led to increased rates of exposure to violence in early life (Atkinson, 2002); socioecological and socio-economic disadvantage which compounds early adversity and are associated with increased health risks (Bellis *et al.*, 2014; Font and Maguire-Jack, 2016); decreased effectiveness of preventive interventions (Blalock *et al.*, 2013); and intergenerational transmission (Chamberlain *et al.*, 2019b; Segal and Dalziel, 2011).

The perinatal period is particularly critical for parents experiencing complex trauma (Marriott and Ferguson-Hill, 2014). Physiological changes can heighten a mother’s trauma responses (Muzik and Rosenblum, 2018). For mothers and fathers, the intimate nature of pregnancy care, birth, breastfeeding and the demands of responding to a young baby can arouse feelings and response patterns reminiscent of their own childhood (Chamberlain *et al.*, 2019b). These experiences can be bewildering as often the original trauma occurred a long time ago and dissociation responses limit insight into underlying causes. Trauma responses can also compound healthcare-related challenges, including engagement with essential services.

Conversely, becoming a parent offers unique lifetime opportunities for recovery and preventing ‘intergenerational cycles’ of trauma (Alexander, 2014). Growing evidence demonstrates how ‘nurturing love’ can positively reinforce parental recovery (Schore, 2019) through a process referred to as ‘earned security’. It is this positive concept that has inspired our project title – *Healing the Past by Nurturing the Future* (HPNF) (Chamberlain *et al.*, 2019c). Becoming a parent is often the first time most people have regular scheduled contacts with healthcare services since childhood, offering opportunities to identify parents at risk and offer support. Despite these opportunities and risks, a national survey reported 98% of perinatal care providers identified trauma as a significant issue affecting Aboriginal and Torres Strait Islander parents; yet almost half (43%) were not ‘satisfied’ with the ability of their service to address trauma (Highet and Goddard, 2014).

There is an urgent need to ensure Aboriginal and Torres Strait Islander parents have access to high-quality perinatal care that is consistent with national trauma guidelines (Kezelman and Stavropoulos, 2012). Understanding the needs of Aboriginal and Torres Strait Islander parents experiencing complex trauma is essential for informing the development of these urgently needed support strategies. This development paper aims to understand Aboriginal and Torres Strait Islander parents’ views to inform trauma-integrated perinatal support. The specific research questions were:

- What things from the past may impact parents now?
- What helps to keep parents strong?
- What are parents’ hopes and dreams?
- What do parents need to achieve these hopes and dreams?
- Are issues previously identified by parents in international studies relevant for Aboriginal and Torres Strait Islander parents?

## Methods

**Figure 1.**
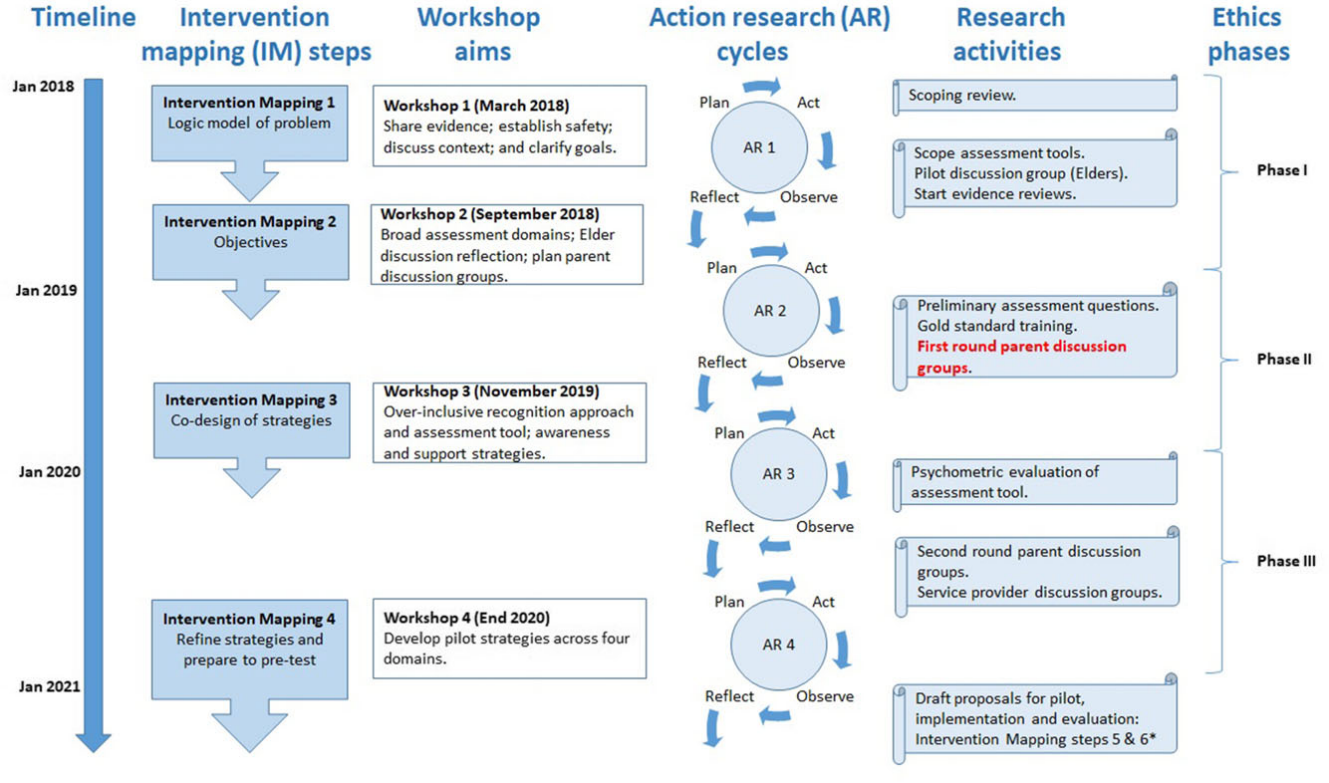
Context of this study within Healing the Past by Nurturing the Future project

The HPNF project uses an ecological, participative Intervention Mapping (IM) approach to co-design acceptable, feasible and potentially effective perinatal awareness, recognition, assessment and support strategies for Aboriginal and Torres Strait Islander parents experiencing complex trauma (Chamberlain *et al.*, 2019c). An overarching protocol for the HPNF project is reported elsewhere (Chamberlain *et al.*, 2019c). Four action research cycles incorporate mixed methods research activities, with reflection and planning stages conducted in four key stakeholder co-design workshops aligned with the first four (of six) IM steps, which are being conducted in the currently funded HPNF project. Future grant proposals will be submitted for IM steps 5 and 6 (implementation and evaluation). This manuscript presents findings from the first round of parent discussion groups as part of the second action research cycle, as shown in Figure 1. We have followed standards for reporting qualitative research in this report (O’Brien *et al.*, 2014).

### Qualitative approach and research paradigm

The HPNF project is structured within an Indigenous research paradigm to centre Aboriginal and Torres Strait Islander parents’ perspectives in the co-design process. Indigenist paradigms recognise ongoing oppression, transgenerational trauma and grief for Aboriginal and Torres Strait Islander communities and aim to assist decolonisation through a process of empowering and privileging Aboriginal worldviews and self-determination (Walter and Suina, 2019). The epistemological position is constructivist/interpretivist, acknowledging multiple realities which must be constructed and interpreted within a social, cultural and temporal context (Santiago-Delefosse *et al.*, 2015).

The specific research study described in this manuscript draws upon foundations of an Indigenist paradigm to also critically engage constructivist grounded theory methods (Mills *et al.*, 2006). As a flexible methodology, and one that is suited to a little explored phenomenon, Aboriginal researchers immersed in data collection and analysis used grounded theory methods aligned with Indigenous research principles (Dudgeon *et al.*, 2020). The research team explored perspectives of Aboriginal and Torres Strait Islander parents regarding trauma-integrated support needs in recovery from complex trauma, and systematically and iteratively generated a theoretical supposition (Chun Tie *et al.*, 2019).

### Researcher characteristics and reflexivity

This research was designed by an Aboriginal-led team of researchers with expertise in public health, midwifery, social work, family health and psychology. Most participants were recruited through partner health services and did not have close personal relationships with the researchers, however, some parents recruited through community networks were known to the researchers. Three authors were immersed in data collection (CC/YC/AH). This approach is consistent with constructivist grounded theory methods and relational Indigenous research methods whereby researchers work in equal partnership ‘with’ study participants to enhance the authenticity and ethical integrity of the process and the analysis and interpretation of the data (Mosselson, 2010).

### Context

Parent interviews and discussion groups were conducted in three Australian jurisdictions, Melbourne, Victoria (VIC), Alice Springs, Northern Territory (NT) and Adelaide, South Australia (SA). Melbourne and Adelaide are metropolitan cities and Alice Springs is a remote town in Central Australia.

Parents were recruited through community networks and partner health services where they attended perinatal care. The contexts from which parents were recruited – maternal, perinatal, community health and hospital services – are heavily biased towards women and our recruitment of predominantly women reflects this. We attempted to mitigate this bias by emphasising that both parents were eligible to participate and ensuring all project documentation referred to ‘parents’ rather than ‘mothers’.

### Sampling strategy

Parents were eligible to participate if they: identified as Aboriginal and/or Torres Strait Islander, were aged 16 years or older, and were currently pregnant or had a child less than 2 years of age. Parents aged 16 or 17 were excluded if they were not considered sufficiently mature by the researcher to understand and provide informed consent or were not willing to ask their own parents to provide parental consent for them to participate. Parents experiencing current serious mental illness were also excluded.

Service providers in partner health services provided information about the study and asked for permission to provide contact details to the study coordinator. Parents could also contact the study coordinator directly via flyers in the clinical areas or community networks. Challenges with engaging parents were expected, given the sensitivities of complex trauma, particularly in relation to parents and the history of forced family separations, and history of inappropriate conduct of research with Aboriginal and Torres Strait Islander people.

### Ethics

HREC applications for the HPNF project were submitted in three phases to enable the development of appropriate safe strategies. HREC approval for this activity was part of ‘phase 2’, and was informed by ‘phase 1’ activities which included discussions with senior Aboriginal women (Clark *et al.*, 2020) and key stakeholders attending a workshop. Three jurisdictional Human Research Ethics Committees (HREC) approved the procedures outlined in this paper. Prior to participation, the study coordinator confirmed study eligibility with parents and obtained informed verbal and written consent.

### Data collection

Data collection methods were piloted and refined with a group of senior Aboriginal women and a workshop with a majority of Aboriginal and Torres Strait Islander participants. Parent interviews and discussion groups were held in August and September 2019. Parents were offered the option of participating in individual interviews or group discussions, at their home or in a family-friendly service setting. Aboriginal researchers facilitated the discussions, which were audio-recorded and later transcribed by a researcher. Data were collected in a non-identifying way and any potentially identifying information (e.g., names) was removed from the original audio transcripts.

Processes to foster safety and empowerment within the interviews and discussion groups included acknowledging the sensitivity of the workshop content, clarifying that participants will not be asked to talk about their own experiences, having a psychologist available throughout the workshop with safety and distress protocols in place (Clark *et al.*, 2020). Other strategies included taking time out when needed, providing a safe quiet space to be used at any time, and the use of mindfulness colouring materials on tables. Visual tools were used to facilitate discussions that built on existing research and international literature; used third-person scenarios to guide a discussion to increase safety and minimise the ‘directness’ and ‘intrusiveness’ of sensitive conversations; captured metaphors and symbolism used by parents to explain complex phenomena; and maintained a ‘strengths-based’ focus on healing. While participants were not asked to speak about their own experiences, many parents were clearly comfortable in sharing their personal experiences as reflected in the findings. The discussion included:

1. Sharing a strengths-based but realistic story of Aboriginal and Torres Strait Islander parents experiencing complex trauma, as a third-person scenario (adapted for each setting) (Ngaanyatjarra Pitjantjatjara and Yankunytjatjara Women’s Council, 2016).
2. Using an adapted ‘Tree of life’ visual approach (Denborough, 2008) with parents to reflect upon the story, and discuss: what impacts upon parents from the past (illustrated as roots of the tree); what keeps parents strong (trunk); parents’ hopes and dreams (branches); and what might help parents achieve their hopes and dreams (leaves). Drawing materials and creative sticky notes (e.g., pebbles, leaves, flowers, fruits, birds, clouds, rainbows) were provided as an aid for parents to share their thoughts and illustrate key messages from the discussions (see Supplementary File 1, e.g., images). This approach has been demonstrated to be safe and effective for discussing trauma in sensitive contexts (Lock, 2016), and has been used previously by the investigators.
3. Sharing coloured cards of themes identified in systematic reviews of parents’ experiences (Chamberlain *et al.*, 2019a; Chamberlain *et al.*, 2019b), and inviting Aboriginal and Torres Strait Islander parents to discuss any additional issues that they felt were important. As well as increasing the comprehensiveness of data, this iterative process improves the triangulation of data sources.

Parents were given a grocery voucher, a small gift (e.g., children’s book) and refreshments to thank them for sharing their expertise. Childcare and transport were provided if needed. For all participants, follow-up well-being phone calls were attempted the following day and week.

### Data processing and analysis

Identifiable recruitment and demographic data were stored on a secure REDCap database separate from study data. De-identified transcripts, photographs of ‘Tree of Life’ drawings and notes were uploaded into NVivo, coded by jurisdiction and a unique discussion ID.

Consistent with constructivist grounded theory methods, we used thematic coding techniques (Green *et al.*, 2007; Mills *et al.*, 2006), including immersion, coding and construction of a ‘core category’ or story. Immersion started with authors involved in data collection, reading the de-identified transcribed data and reflecting on the images and notes. Preliminary open line-by-line coding of de-identified transcripts was conducted in NVivo with two authors, who had been jointly involved in all discussion groups (CC/YC). During coding, co-authors iteratively discussed the meaning and constant comparison to generate preliminary codes, analytical themes and exceptions. Codes were categorised and rearranged using hand-drawn illustrations (Supplementary File 2) and notes to refine analytical themes or theoretical codes and reflect on connections between codes (Mills *et al.*, 2006). A ‘story’ was conceptualised into a theoretical supposition that integrates the main analytical themes that parents shared. Draft findings were discussed with parents to ensure they felt their voice was present in the final text and to include any feedback (Mills *et al.*, 2006).

## Findings

### Participant characteristics

Seventeen parents participated in total across three jurisdictions, in Adelaide (n = 10), Alice Springs (n = 4) and Melbourne (n = 3). Individual interviews were conducted in Alice Springs and Melbourne. In Adelaide, one participant was interviewed individually, and the other nine parents participated in discussion groups (three groups with two parents, and the fourth group with three parents).

Most participants were mothers (n = 15). It was particularly difficult to recruit fathers for this study with only two men participating. The majority of parents were living in major cities (n = 12). Parents reported heritage from both Aboriginal (n = 16) and Torres Strait Islander groups (n = 1), and 16 distinct language groups. Seven parents were familiar with an Aboriginal language. Most parents were over 20 years of age (n = 16) with a mean age of 29 years. More than half were living with a partner (n = 10) and had three or more children (n = 9). The Index of Relative Socio-Economic Disadvantage (IRSD) in this study cohort was similar to the average index, which represents a slightly higher IRSD than the general Aboriginal and Torres Strait Islander population.

### Theoretical supposition emerging from parents’ voices and reflections on the literature

The study’s Indigenist approach, drawing on grounded theory methods, supported the development of an integrated, comprehensive theoretical supposition. Our supposition positions the transformation of the compounding cycle of hurt, as a result of complex trauma, to a reinforcing cycle of nurturing at the intersection of parents’ connectedness, social and emotional well-being and the transition to parenting. Unique opportunities and challenges situated at the interface of these intersecting factors are demonstrated in how they can negatively compound upon or positively reinforce one another. Experiences vary within and between communities. Experiences are also likely to be gendered, recognising the bias in perinatal care towards women and the challenges of recruiting fathers in this study, and poses limitations to this element of our supposition. The following findings reveal the complexity of this work but also demonstrate the opportunities for change when considered through a strengths-based lens, such as being grounded in Aboriginal and Torres Strait Islander parenting practices.

**Figure 2.**
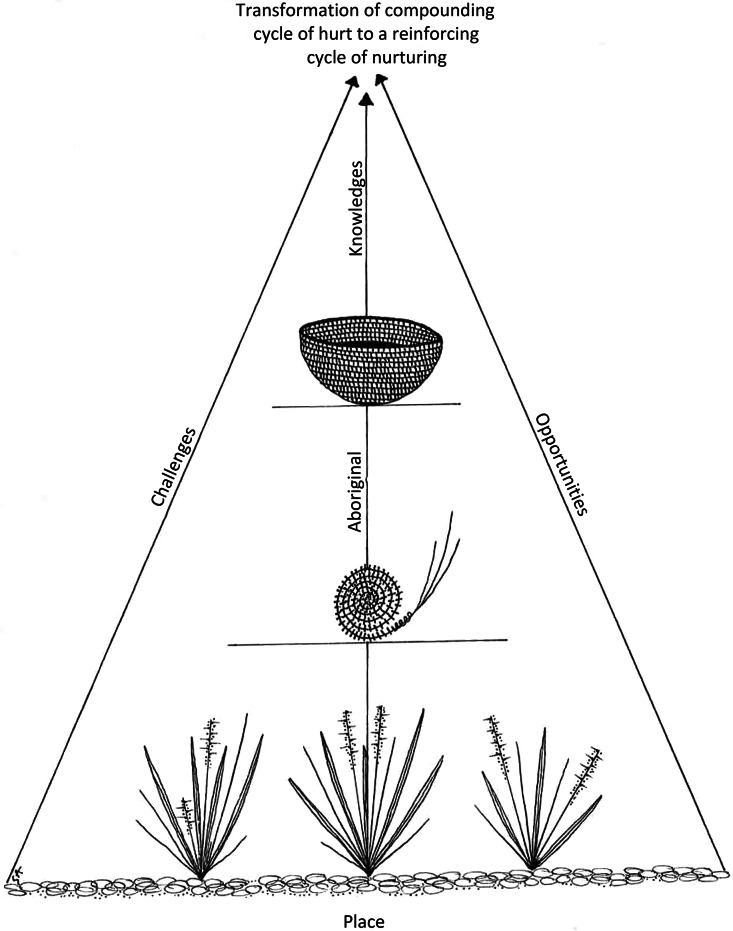
Image representing theoretical supposition

An illustrative description of our theoretical supposition is shown in Figure 2. The grasses, weave and basket represent the cumulative nature of connectedness, well-being and parenting, respectively. Grasses, or reeds, from which baskets are made grow from the country and represent connectedness to place and culture. The weave facilitates the grasses’ transformation into a basket and relies upon generational transferred Aboriginal and Torres Strait Islander knowledges and cultural practices. Without knowing how to weave, a basket cannot be made. Well-being of culture and community kinship networks is represented here. The basket is the product of cumulative knowledges and practices and is only whole and effective when all prior stages are achieved. Aboriginal and Torres Strait Islander knowledges and life challenges and opportunities are intrinsic and external influences that contribute to the sequelae of transition to parenting.

### Main analytic themes

The following **analytic themes** are listed here but, in reality, are interconnected and integral to each other. How they are positioned at the intersection with one another and with Aboriginal and Torres Strait Islander parenting in the context of complex trauma is critical to consider how we can impact upon embedded generational patterns and build positive parenting outcomes for families.

1. **Connectedness** with family, culture, partners and the new baby is central to social and emotional well-being and achieving hopes and dreams for a happy life with their new baby. Disconnection is a root cause of trauma, connection is a core strength and reconnection is an important strategy for recovery.
2. **Social and emotional well-being** is impacted by past trauma, socio-economic challenges and connectedness. Parents use many strategies to keep strong, which is critical for achieving dreams of a happy life with their new baby.
3. **The transition to parenting** brings new hope and dreams for a happy life with their new baby, and connectedness and well-being are central to achieving this. However practical, clinical and cultural support and role models are vital.

**Figure 3.**
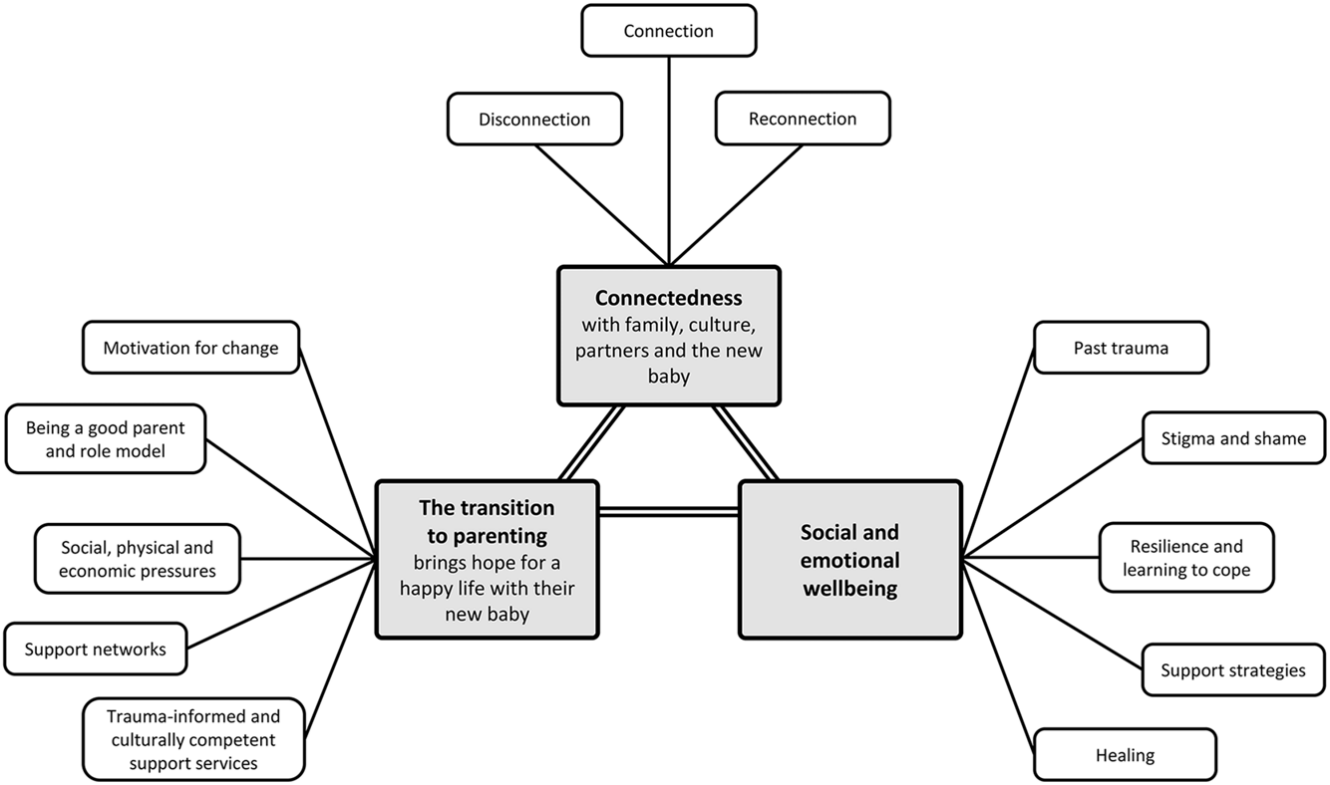
Illustration of analytic themes

We describe parent perspectives (using pseudonyms) under these three analytic themes. An illustration of analytical themes and summary codes is shown in Figure 3.

### Theme 1. Connectedness with family, culture, partners and the new baby

Parents described connectedness as central to social and emotional well-being, recovery and achieving their hopes and dreams for a happy life with their new baby.

Disconnection was identified as a key factor impacting parents in the stories, stemming from colonisation, felt as a lack of belonging, an absence of role models in peoples’ lives, family conflict and as a lack of identity or direction in life.

“My mum’s not Aboriginal and I grew up with her, so we didn’t actually know we were Aboriginal until we grew up because it just wasn’t the thing to speak about.” [Riley]

“Family connections is always such a big thing or even friends…. That’s the thing with Aboriginality forms not everyone knows their whole family. You know they know that they are Aboriginal but not being accepted.” [Riley]

Participants identified the importance of connections, reconnection and trusting relationships in keeping parents strong and achieving dreams for a happy life with their new child. This included connection to country, ancestors and culture, as well as connections with family, friends and “*having someone who loves and cares for them*” [Grace].

“Being on their country, with family, mainly grandmothers…To grow up having that family, strong family connections. Healthy and strong family connections… They probably want their babies to grow up and know their country and culture.” [Grace]

“To learn or reconnect to their culture, especially when you are having a baby, you want to pass that on.” [Carol]

“Culture – which relates to identity and knowing who they are in the world. Not just the bigger world but includes connection to family.” [Rebecca]

Parents described the importance of the relationship with partners in keeping strong. They emphasised new experiences of love and joy that a baby brings into a relationship, and the importance of ‘*having fun*’ (Ella) to foster love, care and bonding.

“They have each other…they can lean on each other if they need to” [Jane and Daniel]

“You know the biggest thing for me is how many people are excited. Like I really didn’t realise that they are just so excited to meet this baby… he is going to have so much love it’s ridiculous even thinking about it.” [Rebecca]

Parents talked about the importance of having compassion for their families and themselves. However, family pressures and obligations and worry about the baby’s safety can pose challenges to reconnecting.

“Compassion, developing compassion for their families too. Because sometimes it is easier to be angry and isolate, and start blaming family. And maybe it is part of the healing process I would have thought, to be angry about the things that didn’t happen, or what happened. And then moving to develop compassion for themselves. They are doing the best job that they could given the situation. We’re all doing the best job that we can, with the knowledge that we have. But compassion. For themselves, and their families and each other.” [Helen]

Parents described things they do to connect with family and culture, including cultural activities, involving Elders and developing cultural guidance for children.

“So I think that therapy is great but I think that we need to look at spirituality as well and how do we make our spiritual connections stronger… Most of my people that I have worked with had no idea what a smoking ceremony was, but then when they have their first their whole perspective on everything changes.” [Carol]

“Eating healthy foods. Hunting with the family. Also, learning song and learning culture from elders.” [Grace]

### Theme 2. Social and emotional well-being

Parents talked about the importance of social and emotional well-being for fostering connectedness and a positive parenting transition, but also highlighted a range of challenges.

Challenges included effects of past family trauma and relationship breakdown, adverse childhood experiences, family disruptions, foster care and institutionalisation - all directly related to colonisation. Parents highlighted feelings of guilt and shame, which related to racism, bullying, stigma and discrimination.

“Colonisation definitely effects…. huge wack shame again, just shame, and shame around being Aboriginal, shame around identity.” [Helen]

“That was one thing I learnt being in a foster home, being from an Aboriginal family, you get treated differently to everyone else, you pretty much get left out… When I was in foster care, that’s when it was the worst. It was horrible. I got pushed to the side, the other kids just got what they wanted. I got hand-me-downs, leftovers. I never got nice things….You sit back and you think, all of these kids that are in care now, what are they going through?” [Sarah]

“Shame, definitely scared to tell people [about trauma history] cause they will think there is something wrong with you.” [Helen]

“I didn’t really like it when I saw the man midwife. … Shame, yes shame…not knowing where to look.” [Jessica]

One father described experiences of discrimination as a male caring for his children. “There are some men with bad behaviour, but at the same time we are all ‘tarred with the same brush’…and I get some strange looks and people ask ‘who’s that’ and I have to say ‘oh that’s my daughter’ – and have to explain that stuff. … You’d bloody kill anyone for her but you know, people see you with a kid and its ‘alarm bells’.” [Jordan]

Parents identified specific impacts related to trauma, including grief and loss, symptoms of avoidance such as substance use, challenges with managing distress and not learning how to cope. These were associated with feelings of helplessness and lack of confidence. Parents also highlighted complexity and comorbidity, health issues and physical and mental health problems.

“I had to stop all my anxiety meds and that is really hard and having to not even drink. I don’t think people realise how hard that is especially when you have a trauma background and you’re using some of that stuff that as a crutch. I struggled, and the only think that kept me going was that I don’t want anything to happen to my kid.” [Carol]

“Confidence, everybody lacks so much confidence, that’s why they don’t want to put themselves out there and talk about things.” [Kate, Melissa and Nicole]

“Yeah, it’s not always what’s on the outside, really dig into what’s into the inside and really try and get those underlying issues out.” [Riley]

For keeping strong, parents highlighted the importance of resilience, and survival, learning about trauma, good health and being able to look after themselves. Many parents described a ‘fear of repeating the past’ and the need to address trauma to avoid passing trauma onto their children.

“sometimes you know trauma can be a gift in a way, look at it as a learning experience, as a learning curve, you get all the hard stuff out of the way earlier and you know what you don’t want to do and how you don’t want to do it. I guess you are learning from the mistakes of others.” [Carol]

“I don’t want my kid to go through anything I’ve been through… Generally people want to create a disconnection to the generational trauma so it doesn’t affect their children and their babies.” [Riley]

“To feel like they won’t pass on their childhood trauma.” [Nicole]

Other strategies included being strong and positive, feeling in control and empowered. This could be helped by being valued and acknowledged by others, to build self-esteem confidence, reassurance from others. Continuing to do the things they enjoy, taking time out and self-care were also seen as critically important to social and emotional well-being.

“I think that we need to put a positive spin on it - you know that trauma shouldn’t be negative. Strengths, like look at how much strength you’ve got to be able to come through all of that, you are doing an amazing job. There are so much negative words around everything. But I think most of the time you need to be told that you are doing really good. Everyone needs to be told that they’re really proud of what they’re doing, and that everything’s going to be ok and that you know, you’re not your past, you’re not your trauma, that doesn’t define you. A lot of acknowledgement is good too…they tell me all the time what a good job I’m doing, otherwise I wouldn’t know” [Carol]

“Self-care, that’s what people forget to do is put their self and their family before others.” [Kate, Melissa and Nicole]

“‘People just assume that you are running on full batteries all the time – but you’re not, and just having those moments where you can have like a little bit of that spark and stuff to do whatever it is that helps you feel that spark. Maybe it is just having an afternoon, or a bath, or to read a book, watch your favourite soapie or trash tv …practising self care, taking care of yourself and baby, healthy lifestyle, importance of looking after yourself and baby.” [Rebecca]

In discussing things that help parents achieve their goals in relation to social and emotional well-being, parents discussed a range of expressive strategies, including art, cultural therapies and developing emotional skills.

“I just think that talk therapies are not good for Aboriginal people. I think if you were going to do art therapy … Why aren’t we doing big smoking ceremonies and having our Elders. Especially during pregnancy, how good would it be to have a renowned Elder there and to have the women smoked out while they’re pregnant.” [Carol]

“Like writing things down like a vision board, write down how you’re feeling, releases it and not hold on to all your feelings.” [Melissa]

“Because it is an outlet for frustration, anger and human – creativity is a good feeling, art, we feel through our art and paint our stories out, and even music it is something that is passed on.” [Helen]

Many parents felt it was important to speak up about trauma and to be able to get help or talk to someone. Parents discussed several counselling strategies that could help, including group counselling, holistic family therapy, relationship counselling, drug and alcohol counselling and Eye Movement Desensitization and Reprocessing (EMDR).

“Therapy, group, family or individual, because then you get to experience that powerful group that you know rather than just an individual that is loaded with shame. And other people have similar experiences, and that connection. How much does connection smash out all this negativity stuff.” [Helen]

“You look at our culture, we dance, you know we sing with our trauma, we dance, we play instruments, you know it’s a lot of movement to deal with our stuff. Of course stuff like EMDR is going to work better for us than talk therapy.” [Carol]

Parents acknowledged the importance of acceptance, forgiveness and allowing time to heal in recovery from trauma.

“Allowing time to heal, that’s hard.” [Helen]

“Acceptance and forgiveness. To be able to accept what’s happened and move on. Otherwise you just don’t go nowhere if you can’t accept and move on. You just stuck in the same.” [Kate, Melissa and Nicole]

“Forgiving yourself is not necessarily having to have that particular person be a part of your life. …I am forgiving you so that I don’t harbour anymore bad feelings because that is bad for me, so it’s like healing. I am not allowing you to come back into my space, and create the same situation as before.” [Rebecca]

### Theme 3. Parents hopes and dreams for a happy life with their new baby

Becoming a parent is a time of hope, and both connectedness and social and emotional well-being can help to positively reinforce recovery from complex trauma. Conversely, parents identified challenges that could hinder the capacity to achieve dreams of a happy life with a new baby.

Parents talked about hopes for a healthy baby and having the baby home with them, being the best parents they can be and giving their baby the best life they can.

“People want happy healthy babies – people ask me if I want a boy or a girl, and I say I don’t care as long as they are healthy! People are more aware of wanting children to have the best start at life – whatever that looks like.” [Rebecca]

“Like a new life, give the baby the best life that they can give…A good life for the baby.” [Riley]

Parents highlighted new opportunities that starting a family brings. Becoming a parent can provide a sense of responsibility, purpose and motivation for change. Several parents saw parenting as an opportunity for recovery and posttraumatic growth.

“I agree that becoming pregnant and the decision to keep the baby gives people a sense of responsibility and purpose.” [Carol]

“I think the baby is keeping them strong too, something else to focus on something that is not yourself anymore, sort of puts things in perspective. In a lot of ways it makes or breaks you.” [Carol]

“Once you have your first child it’s a completely different life for some or most.” [Kate, Melissa and Nicole]

Many parents talked about being a ‘good’ parent, which included learning to relate to the child, wanting to be emotionally responsive, and being a good role model. Some parents talked about idealistic notions of trying to be a ‘perfect’ parent.

“Just being a good mother, being good parents.” [Riley]

“I think a good parent is someone who is there for their kids, who encourages their kids, who teaches their children and that whole feeling of just feeling safe, I think that is very important.” [Carol]

“That was one of the other things that came up for parents that have had that more kind of challenging upbringing, the pressure to be perfect, probably more than people that haven’t had those challenges.” [Rebecca]

Parents identified challenges to achieving their hopes and dreams, such as the changing roles of parenting, conflicting feelings about being pregnant, being ready and able to parent and the importance of learning about parenting – but in a culturally appropriate way.

“I struggled being pregnant, I wasn’t one of those happy pregnant people. I couldn’t picture my baby. I knew that there was something inside me, but I had no idea what that meant so I really struggled….I think if you were going to have a parenting group it would really have to be run by Aboriginal people, because then you’ve got White fellas telling Black fellas how to raise their kids and that’s not going to work.” [Carol]

Parents highlighted social, physical and economic pressures that can have an ongoing impact on achieving their parenting aspirations, including their home environment and instability, employment challenges (impacted by lack of education), poverty and drug and alcohol use, which can lead to involvement with the justice system.

Overcoming socio-economic challenges was an important strategy for enabling a happy and satisfying life, including work to support family, a stable home and getting a good education for their children. Staying away from drugs and alcohol and staying out of trouble were also seen as critical to achieving these aspirations.

“To get a good job to support each other.” [Sarah]

“A stable home.” [Isabelle and Rose]

“They probably want them to go to school and get their education, both ways.” [Grace]

“I think housing is one of the biggest things that parents need, and I know that there is huge shortfall. But if you don’t have a house, or somewhere to be, I mean that’s your foundation and if you don’t have that how can you build anything else around it. So I really think housing is always going to be the number one, because you can’t have anything without that first.” [Carol]

“they sent me to a special program. I learnt anger management skills and how to look for work at the same time. So, working on myself and employment at the same time. Pathway program - they taught us how, shopping, to help with finance, how to cook in the kitchen.” [Jane and Daniel]

Lack of support, loneliness and having no one to talk to were also seen as major challenges for parents with a new baby. Support networks – including family support, friends and being able to choose a support network that is appropriate (e.g., age- and gender-appropriate peer support groups) are critical.

“I feel like, bit lonely you know. I’m happy, but sometimes I get lonely to go back to mum’s, so I go back there…but it’s noisy. It’s like so many kids are there…six kids in the house. Crazy. [Mum] helps me. I can try to learn to look after kids on my own. But mum said you need a bit of extra help too.” [Jessica]

“Supporting each other as brother and sister. I think it’s that whole concept of ‘it takes a village’ and in my experience that is definitely the case…” [Rebecca]

“Some families, some extended families, they don’t have the support. It’s hard for them without any support. So they can’t make a better life because they haven’t got anyone there to support them. But with that extra support, you can get a long way. It doesn’t have to be family. You can have friends that are more like family than friends. Some people don’t have that luxury. Parents can have trouble identifying what support they do have.” [Jordan]

Parents also discussed the role of internet information and networking for enabling connections.

“Don’t lose your friends, because at the end of the day they’re the ones who are going to pick you up.” [Riley]

“My niece she looks for help and support…Especially the social networking. She knows a lot of other mummies. Her friends are mums.” [Jane and Daniel]

However, participants discussed how setting healthy boundaries with children and family is also important for achieving a happy family life and well-being.

“You have your family and then you take on other people’s things and then it affects your family, so you need to have your boundaries.” [Kate, Melissa and Nicole]

Participants highlighted the importance of health and community services in helping them to achieve their hopes and dreams, but discussed the challenges of receiving perinatal care. Fear of disclosure and concerns about child protection were identified by parents as a barrier to seeking support.

“Staying connected to services that will help and show good impact to them and their baby.” [Isabelle and Rose]

“Yeah I was triggered in the hospital with some of the procedures… And then when I told them that I’d been triggered. Then they’re like we don’t want to have to do this to you, and I was like well what choice do I have. Which is you know feelings of guilt and shame, even the hospital that’s how you feel.” [Carol]

“They need to feel safe. I don’t even know how to do that, I always struggle with that honestly. Because within the institution, and mandatory reporting. How can you heal something when you are fearful of telling someone about it.” [Helen]

Parents identified things that may help to improve access to support services, including parents accepting that they need help, having role models and motivation to access services, realising what support is available, knowing where to access support and having agency to access support when needed. However,

“Well you have to have motivation to want to do this and want to do that and be able to deal with all that. Have to be motivated to want to go find a service, to make appointments, to go to the appointments.” [Nicole]

“I think a lot of people know what they want but they not going to ask for it. They need to learn to ask.” [Kate, Melissa and Nicole]

Parents highlighted the need for compassionate and culturally competent care and good communication. Trauma-informed Aboriginal health services, workers and liaison officers and support workers who can be trusted are critical for supporting navigate challenges. Training and support for workers, continuity of care and female care providers were also seen as important.

“Definitely mindful of the language that they use. Like asking before any procedures. like asking, before they do the examination and stuff, are you ok here, are you comfortable, and then just letting them know that as soon as they don’t feel comfortable that the procedure can just stop.” [Rebecca]

“Yeah, I’m going through the Aboriginal birthing program. I’ve got a really good relationship with the midwife.…. It’s a really good program.” [Riley]

“I think there needs to be more of a focus on training. …why aren’t we focussing more on training up Aboriginal people.” [Carol]

Support for fathers was identified as a major gap in current support services, as recognised by both mothers and fathers.

“Most supports are aimed at mothers. From a male perspective, we feel we are behind the eightball a bit. Services are very maternity focussed, and men are excluded. …Most parent groups are all mothers and we really out of place. Town people they don’t care – they are more relaxed being around women and in mixed groups, but [us] country people are totally different…But have specific groups, and then everyone feels safe and feels good.” [Jordan]

“A lot of fullas don’t know where to gain access to do that” [Kate, Melissa and Nicole]

## Discussion

Seventeen parents, predominantly mothers, described a range of factors illustrating the interdependence of connectedness and physical, social and emotional well-being at the interface of the parenting transition in the context of complex trauma. At this interface, intrinsic and external influences can negatively compound existing trauma or positively reinforce recovery and achieving dreams for a fresh start with a new baby. Experiences vary within and between communities. Challenges recruiting fathers for this study illustrate the gender bias in early parenting support.

Aboriginal and Torres Strait Islander parents reinforced many findings identified in a review of qualitative studies involving parents who have experienced maltreatment in their own childhoods (Chamberlain *et al.*, 2019b). However, in this current study, Aboriginal and Torres Strait Islander parents placed greater emphasis on the centrality of connectedness to experiences of trauma; keeping parents strong and helping parents to achieve their dreams of a happy life with their new child. These findings are consistent with those reported in a previous study involving Victorian Koori parents (Gee *et al.*, 2020).

Study strengths include the use of Indigenist approaches and the relational authenticity of research which is Aboriginal-led, conducted and interpreted by Aboriginal interviewers and a predominantly Aboriginal authorship team with connection to each of the jurisdictional contexts. A limitation of the study is the lack of fathers recruited to participate. This reflects the difficulty of engaging men in perinatal care services, as clearly voiced by fathers in this study.

### Implications for practice

There is a critical need for acceptable, effective, culturally-safe, trauma-integrated care for Aboriginal and Torres Strait Islander parents, to disrupt intergenerational cycles of historical and complex trauma. There are several critical components of trauma-integrated perinatal care. First, there is a need to increase parent and service provider awareness of complex trauma experiences and responses that may occur during care and the transition to parenting. As many people do not disclose a history of abuse or trauma until later in life, ‘universal precautions’ that foster safer practices for all parents are needed (Coles and Jones, 2009). Second, while helping parents to identify complex trauma and recovery strategies is vital, *how* those sensitive discussions are held is essential to ensure that the benefits of any assessment outweigh any harms (Chamberlain *et al.*, 2020). Trauma should be conceptualised as understandable protective responses to adverse experiences, consistent with the Power Threat Meaning Framework (Johnstone and Boyle, 2018), to avoid pathologising parents.

Finally, parents highlighted the importance of a broad range of support strategies that go beyond counselling approaches to include practical, social and financial support, creative expression therapies and peer support. Participants in this study highlighted the importance of support, particularly where trauma has resulted in a disconnection from family and other community networks. But *how* support is provided is critical. Support should be ‘hope-inspiring’, strengths-based, safe and nurturing to harness the unique opportunities for recovery during this critical time (Muzik *et al.*, 2013). Importantly, parents highlighted the need for having fun and celebrating the joy children bring as central to parenting support and nurturing recovery from complex trauma. Parents identified a need for culture to be integral to support, and using less direct, culturally-informed approaches to learning, such as cultural role models and ‘educaring’ (Atkinson, 2008). This is consistent with cultural approaches to supporting new parents with support from mothers and ‘Aunties’. These indirect strategies are safer and can help parents to build confidence and skills at their own pace in a gentle, empowering way that minimises feelings of shame and inadequacy. There is an urgent need for specific gendered support and for perinatal care services to be more inclusive of fathers.

### Implications for policy

Practical supports such as housing, employment opportunities, sufficient finances to live with dignity and legal support is critically important to enable parents with complex trauma to achieve their hopes and dreams of a happy life with their new baby. These findings are consistent with calls for a comprehensive multilateral policy approach for Aboriginal families to improve equity (Arabena, 2014). Investment at this critical interface of the parenting transition will directly improve parental social and emotional well-being, with compounding positive impacts on parents’ capacity to care for their baby and ‘reconnect’ with vital networks (Gee *et al.*, 2014). In contrast, continued failure to invest at this critical parenting interface is likely to have a negative compounding intergenerational effect, as currently illustrated with the tragic compounding rates of Aboriginal and Torres Strait Islander family disruptions and intergenerational trauma (O’Donnell *et al.*, 2019).

Perinatal care providers are uniquely placed to work with communities to identify and facilitate support for Aboriginal and Torres Strait Islander parents experiencing complex trauma. The skilled and diverse perinatal workforce employs a wide range of highly trained medical professionals (e.g., obstetricians, perinatal specialists, neonatologists, anaesthetists), nurses with postgraduate midwifery qualifications, Aboriginal and Torres Strait Islander health workers with specialist antenatal skills and mental health professionals (e.g., psychiatrists, psychologists, family support workers, social workers). Despite this extensive expertise, perinatal providers currently lack confidence in the ability of their services to address the needs of Aboriginal and Torres Strait Islander parents experiencing trauma (Highet and Goddard, 2014). This is a major gap which poses an increased risk that parents may be inappropriately referred to child protection services, potentially causing more harm and compounding trauma. We acknowledge the complexity of providing effective support for parents experiencing complex trauma. Midwives have provided compassionate and skilled support during this critical parenting transition for millennia (Chamberlain *et al.*, 2016). There is an urgent need to reclaim ‘wise counsel’ models of care that can address complexity by utilising the best available evidence and community expertise to support Aboriginal and Torres Strait Islander parents to recover from the legacy of intergenerational trauma.

### Implications for research

The greater focus on connectedness highlighted by Aboriginal and Torres Strait Islander parents in this study, as both an essential source of trauma and element of recovery, reinforces the need for any assessment tool for complex trauma to incorporate Aboriginal and Torres Strait Islander conceptualisations of social and emotional well-being (Chamberlain *et al.*, 2019c).

There is an urgent need to develop, implement and evaluate gendered, culturally appropriate, safe and effective models of trauma-integrated care for Aboriginal and Torres Strait Islander parents experiencing complex trauma. We suggest these would be best developed in collaboration with services that have cultural expertise (e.g., birthing on country models) (Hickey *et al.*, 2018; Marriott and Chamberlain, 2019), and incorporate flexible approaches to enable ‘diffusion of innovation’ across the sector (Greenhalgh *et al.*, 2004). Given the critical period of vulnerability for the infant, any effective strategies to promote nurturing care are likely to be highly cost-effective, regardless of the degree of intensity of parental support required (Britto *et al.*, 2017). Economic evaluations that include child, parent and family benefits from effective support are likely to demonstrate significant cost-savings across multiple sectors in both the short, medium and long term. Economic evaluation is needed to promote sustainability of programmes, which is particularly critical in Aboriginal and Torres Strait Islander health where many programmes are funded for limited periods, with a potential ‘net-negative’ impact for families and service providers affected by constant ‘program cycle’ fatigue.

## Conclusions

The transition to parenting presents a unique opportunity to support Aboriginal and Torres Strait Islander parents to improve connectedness and social and emotional well-being, and transform intergenerational ‘cycles of trauma’ into positively reinforcing ‘cycles of recovery’ in achieving their hopes for a happy life with their new baby. Perinatal care providers are ideally placed to build on existing expertise within the sector to develop compassionate, skilled and wise models of care. There is an urgent need to consider gender bias and the needs of fathers within perinatal care, and to develop and evaluate acceptable, effective models of culturally appropriate trauma-integrated support for Aboriginal and Torres Strait Islander parents.

## Supplementary material

For supplementary material accompanying this paper visit http://dx.doi.org/10.1017/S1463423621000463. click here to view supplementary material
