# Supplementary figures and images for: Healing the Past by Nurturing the Future: Aboriginal parents’ views of what helps support recovery from complex trauma: Indigenous health and well-being: targeted primary health care across the life course
Source: Prim Health Care Res Dev. 2021 Sep 30;22:e47. doi: 10.1017/S1463423621000463 (PMC8506449; doi:10.1017/S1463423621000463)

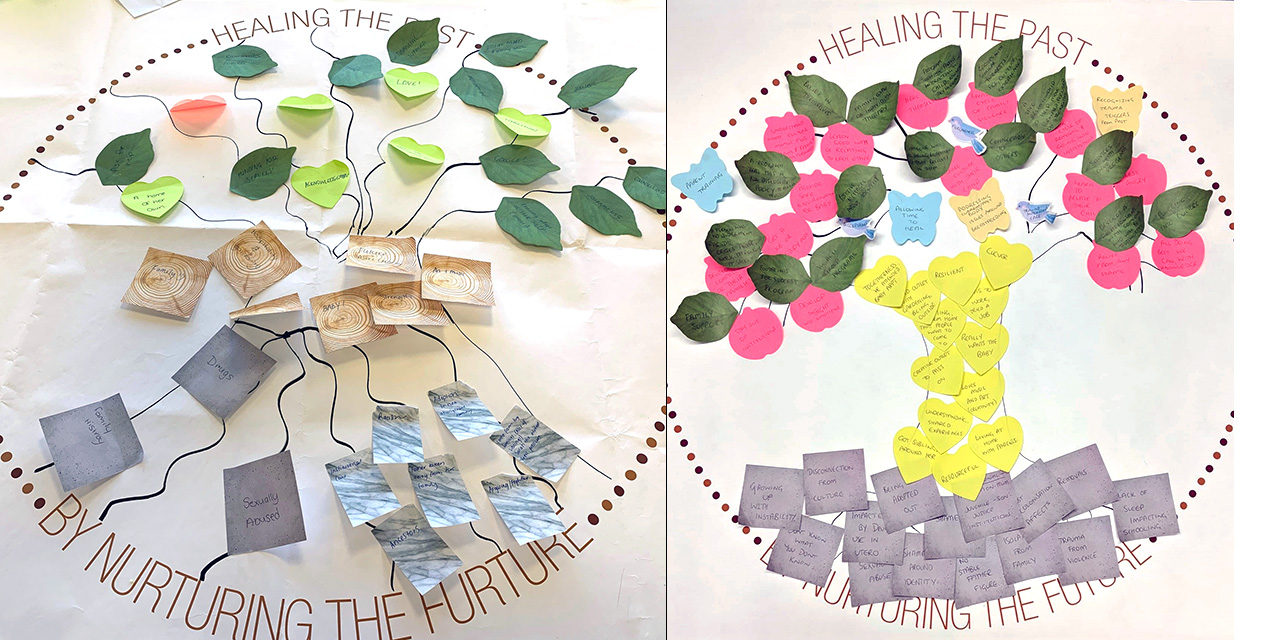

Supplement: Supplementary file 1 [file phcsup.zip › S1463423621000463sup001.jpg]

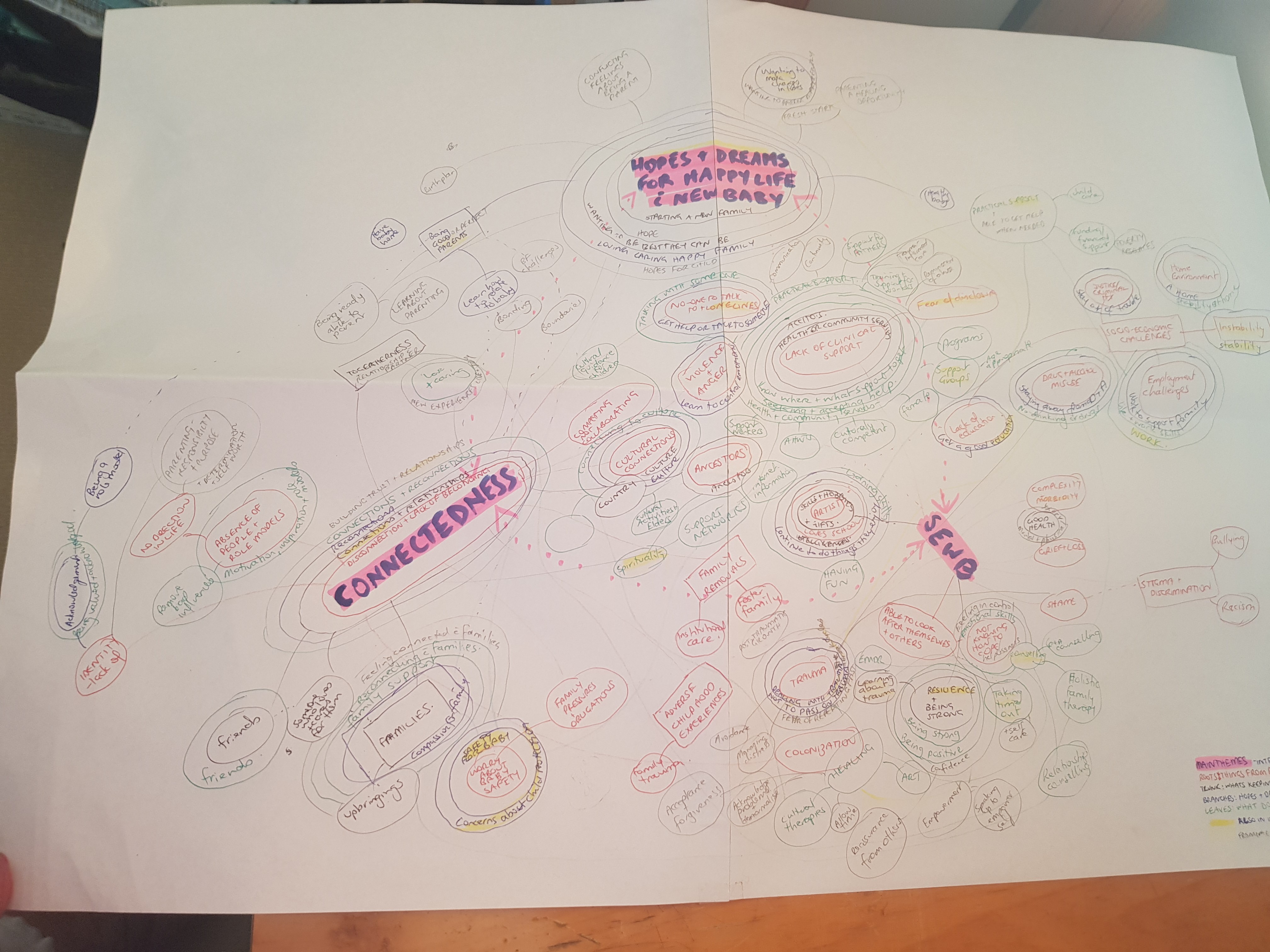

Supplement: Supplementary file 1 [file phcsup.zip › S1463423621000463sup002.jpg]
